# DSL_child_-Algorithm-Based Hearing Aid Fitting Can Improve Speech Comprehension in Mildly Distressed Patients with Chronic Tinnitus and Mild-to-Moderate Hearing Loss

**DOI:** 10.3390/jcm11175244

**Published:** 2022-09-05

**Authors:** Benjamin Boecking, Leonie Rausch, Stamatina Psatha, Amarjargal Nyamaa, Juliane Dettling-Papargyris, Christine Funk, Kevin Oppel, Petra Brueggemann, Matthias Rose, Birgit Mazurek

**Affiliations:** 1 Tinnitus Centre, Charité-Universitätsmedizin Berlin, 10117 Berlin, Germany; 2 Terzo Institute, ISMA AG, 96515 Sonneberg, Germany; 3 Medical Department, Division of Psychosomatic Medicine, Charité-Universitätsmedizin Berlin, 10117 Berlin, Germany

**Keywords:** auditory training, hearing aids, mild-to-moderate hearing loss, tinnitus-related distress, psychological epiphenomena

## Abstract

Background: Patients with chronic tinnitus and mild-to-moderate hearing loss (HL) can experience difficulties with speech comprehension (SC). The present study investigated SC benefits of a two-component hearing therapy. Methods: One-hundred-seventy-seven gender-stratified patients underwent binaural DSL_child_-algorithm-based hearing aid (HA) fitting and conducted auditory training exercises. SC was measured at four timepoints under three noise interference conditions each (0, 55, and 65 dB): after screening (t_0_; without HAs), HA- fitting (t_1_), additional auditory training (t_2_), and at 70-day follow-up (t_3_). Repeated-measure analyses of covariance investigated the effects of HAs (t_0_–t_1_), auditory training (t_1_–t_2_), and the stability of the combined effect (t_2_–t_3_) on SC per noise interference level and HL subgroup. Correlational analyses examined associations between SC, age, and psychological indices. Results: Patients showed mildly elevated tinnitus-related distress, which was negatively associated with SC in patients with mild but not moderate HL. At 0 dB, the intervention lastingly improved SC for patients with mild and moderate HL; at 55 dB, for patients with mild HL only. These effects were mainly driven by HAs. Conclusions: The here-investigated treatment demonstrates some SC-benefit under conditions of no or little noise interference. The auditory training component warrants further investigation regarding non-audiological treatment outcomes.

## 1. Introduction

Tinnitus has been defined as “the conscious awareness of a tonal or composite noise for which there is no identifiable corresponding external acoustic source” [1]. Prevalence estimates vary widely and range from 5% to 43% [2,3]. Risk factors for the tinnitus sound are heterogeneous [4] and—depending upon the proportion of peripheral vs. central contributions in a given patient population—can, but do not have to, involve hearing loss (HL) [4,5,6]. Both chronic tinnitus and HL have been linked to difficulties with speech comprehension (SC), especially in surrounding noise [7]; however, not unequivocally so [8]. Clinical management of HL—with or without concurrent chronic tinnitus symptomatology—aims to ameliorate HL by means of hearing aids (HAs). Whilst HA technology tends to improve individuals’ listening ability [9,10], its benefits to tinnitus-related distress (TRD) [11,12] and SC are mixed [13,14]. Possible interacting influences include non-specific higher-level (e.g., age [12,15]), neuropsychological (e.g., working memory capacity [16], attentional control [17]), or affective phenomena (e.g., mood [18,19], anxiety [20,21]). 

Similarly, research investigating the effects of auditory training on HL or SC suggests only small improvements across outcome domains such as speech intelligibility, cognition [22], or self-reported hearing ability [23,24], although study results are sparse and mixed to date. By contrast, benefits of auditory training to TRD have been documented [25]; though not been tested in sufficiently powered high-quality studies [26]. 

Combined effects of HAs and auditory training for adults with mild-to-moderate HL are underinvestigated. A recent study reported that a 21-day hearing therapy, which consisted of binaural HA fitting and a CD-enhanced 14-day self-study auditory training program, was associated with significant reductions in TRD in a sample of *N* = 177 patients with chronic tinnitus and mild-to-moderate HL [27]. The current study expands this investigation by examining this intervention’s effect on patients’ SC in silence and at varying degrees of noise interference. We hypothesised that the intervention would significantly (after the intervention) and lastingly (at 70-day follow-up) improve patients’ SC given no (0 dB), medium (55 dB), and high (65 dB) noise interference. Using exploratory analyses, we further investigated (a) whether any putative effects were attributable to HAs, auditory training, or both components and (b) associations between SC (improvements) and psychological variables that were obtained at baseline (TRD, depressed mood, anxiety, perceived stress, and psychological distress). The latter investigations were non-directional, as both detrimental and beneficial effects of emotional arousal on SC appear reasonable (e.g., [28,29]).

## 2. Materials and Methods

### 2.1. Participants and Design

Between 2017 and 2019, *N* = 177 patients with chronic tinnitus (52.4% female; age_mean_ = 59.61 years; *SD* = 7.46; age_median_mild_ = 58.50; age_median_moderate_ = 63.50) participated in this study. All participants were informed about the scope as well as aims of the study and signed informed consent agreements. The Charité Universitaetsmedizin Berlin’s ethics committee approved the study (EA1/114/17). All principles of the Declaration of Helsinki were followed. See Table 1 for an overview of patient characteristics (note that the sample was identical to the current study’s predecessor paper [27]). For further information on the study design and research protocol, including power analysis and randomisation procedures, the reader is referred to [27]. 

Upon inclusion, all participants completed a set of screening measures (timepoint t_0_), the results of which are reported elsewhere [30]. Applying a cross-over design, participants were then randomised to an immediate (IIG) or delayed intervention group (DIG). The IIG group completed the intervention (HA fitting + auditory training) immediately after randomisation, whereas the DIG group completed it following an initial waiting period (timepoint_wait_—DIG only). 

Within 14 days after screening, participants in each group received HAs (timepoint t_1_), with t_0_–t_1_ differences thus reflecting putative effects of HAs on SC. Following a one-week adjustment period, participants completed daily auditory training exercises over 14-days (timepoint t_2_), with t_1_–t_2_ differences thus reflecting putative additional effects of the auditory training on SC. The stability of the combined effect of HAs and auditory training was examined after a 70-day follow-up period (timepoint t_3_), with t_2_–t_3_ differences thus reflecting the stability of the overall intervention effect. 

**Figure 1:**
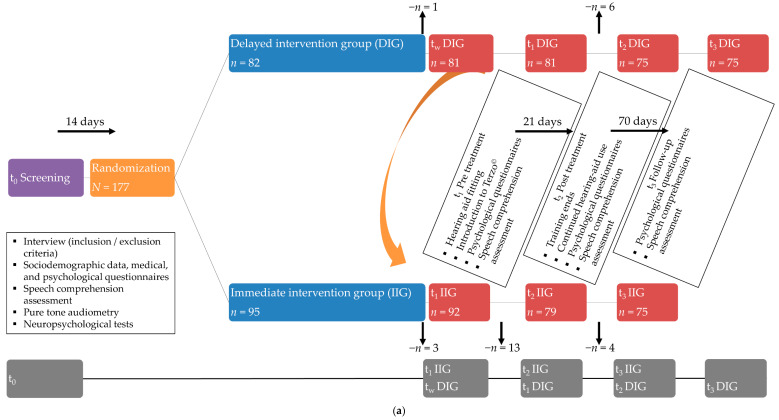

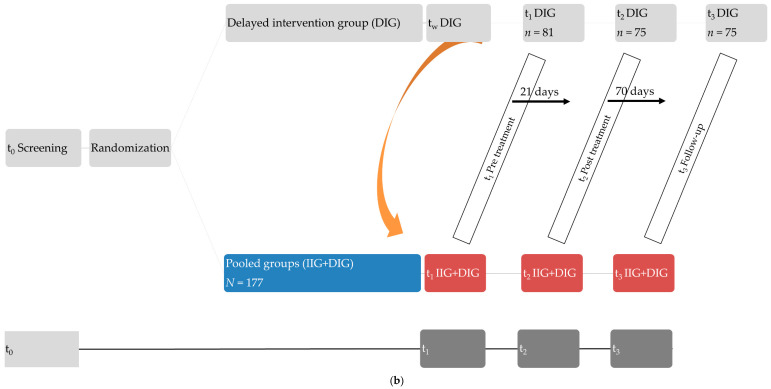
(**a**) Overview of the randomised, controlled cross-over design, measurement timepoints, and dropouts. IIG = immediate intervention group; DIG = delayed intervention group; t_1_ = pre-treatment; t_2_ = post-treatment; t_3_ = follow-up; t_w_ = waiting timepoint (DIG only). Dropout rates are indicated for each arm and measurement timepoint; (b) within-subject analyses investigating treatment-related effects in the pooled sample as applied in the present study. Pooling for (**b**) was possible because the IIG and DIG did not differ on any of the investigated outcome measures at screening (t_0_) or pre-treatment (t_1_IIG; t_1_DIG).

SC measures were obtained alongside potential influencing factors at each of these timepoints (t_0_: without HAs; t_1_, t_2,_ and t_3_ with HAs) under three noise interference conditions each (0, 55, and 65 dB). Participants’ hearing ability was measured once at t_0_ using standard pure tone audiometry. Because no measurements were obtained at the waiting timepoint (timepoint_wait_; DIG only), all analyses were conducted using the pooled total sample (IIG + DIG). Pooling was possible, because (a) the IIG and DIG groups did not differ on any outcome variable at t_0_ and t_1_, (b) there were no group × time interactions across all timepoints (i.e., the overall longer time period in the DIG group, compared with the IIG group, did not exert an effect on the investigated outcomes), and (c) neither ‘previous HA use’ nor ‘duration of HA-use during the intervention’ differed between groups (cf. [27]). See Figure 1 for an overview of the design (adapted from [27]).

#### Hearing Therapy

Terzo^©^ hearing therapy [31] was originally developed for patients with HL. It combines specialised Terzo^©^ HA fitting with an auditory training component. As previously reported in [27], subjects were first provided with a brief educational counselling following which they underwent binaural HA fitting. HAs were adjusted to participants’ individual levels of HL and used the *Desired Sensation Level, v5 child algorithm* to maximise speech audibility in real-world settings [32,33]. Ear moulds were used routinely, and language-specific adaptive parameters were largely deactivated. During the intervention period, HAs were worn for 9.26 h/day, *SD* = 4.14. During the follow-up period, for 9.49 h/day (*t*[145] = −0.48, not significant).

Following an adjustment period of one week, participants independently completed a standardised auditory training program for approximately 1 h/day for 14 days. The auditory training comprised a combination of auditory materials (CDs) and workbook-based exercises, which included comprehension tasks pertaining to numbers, texts, similar-sounding words or syllables. All exercises required mnestic and concentration abilities. Thematic blocks included (1) SC with and without noise, (2) concentration, (3) acoustic retention, (4) semantic memory, and (5) acoustic crossword puzzles. A training manual featured sequential exercises that were linked to the specific days of the intervention period. Participants could record their progress in daily protocol sheets. After the intervention (t_2_), participants returned the training CDs. HAs and self-instruction materials were returned at the 70-day follow-up (t_3_).

### 2.2. Measures

#### 2.2.1. Speech Comprehension

Speech comprehension was measured with an adapted version of the Freiburger Sprachtest [34]. Here, 20 words were frontally presented at 65 dB—in silence or with 55 dB or 65 dB speech babble noise interference. Noise interference was broadcast using spherical ceiling speakers or two speakers from opposite directions. SC was operationalised as the proportion of correctly identified words per noise interference condition (%).

#### 2.2.2. Hearing Ability

Participants’ hearing ability was assessed using standard pure tone audiometry. Here, patients indicated the quietest detectable sound (dB) across eight frequency ranges from 250 to 10.000 Hz. Mild HL was defined using a hearing threshold of 20–40 dB; moderate of 41–60 dB [35].

#### 2.2.3. Tinnitus-Related Distress

Tinnitus-related distress was measured by three self-assessment questionnaires: the Tinnitus Questionnaire (TQ, German version; [36]), the Tinnitus Handicap Inventory (THI, German version; [37]), and the Tinnitus Functional Index (TFI, German version; [38]). Additional information can be found in [27].

#### 2.2.4. Psychological Epiphenomena

Psychological epiphenomena, namely perceived stress, anxiety, depression, and psychological distress, were assessed with the Perceived Stress Questionnaire (PSQ; [39]), the Hospital Anxiety and Depression Scale (HADS, German version; [40]), and the ICD Symptom Rating (ISR; [41,42]).

### 2.3. Statistical Analyses

We conducted separate sets of analyses for patients with mild (HA_mean_ ≤ 40) and moderate HL (HA_mean_ > 40).

Following descriptive analyses, Pearson correlation coefficients *r* investigated (a) associations between participants’ age, hearing ability, psychological distress and SC at screening (t_0_); as well as (b) associations between psychological distress at screening and *change* in SC (between t_0_ and t_1_, t_1_ and t_2_, and t_2_ and t_3_). Correlation coefficients were compared for patients with mild vs. moderate HL using MedCalc (https://www.medcalc.org/calc/comparison_of_correlations.php, accessed on 1 August 2022) and interpreted according to Cohen (*r* ≥ 0.10 = small effect, *r* ≥ 0.30 = moderate effect, *r* ≥ 0.50 = strong effect, [43]).

To identify the effects of Terzo^©^ hearing therapy on SC, we computed three (0, 55, and 65 dB noise interference) × two (mild vs. moderate HL) repeated-measure analyses of covariance (rmANCOVAs) with ‘SC’ as a four-level outcome factor (t_0_: without and t_1_,t_2_, and t_3_ with HAs) and ‘time since screening’ (that differed for participants in the IIG vs. DIG group; days) as well as ‘age’ (years) as covariates. Partial *η²*s estimated effect sizes (<0.06 = small effect, 0.06–0.14 = medium effect, >0.14 = large effect). Post hoc comparisons used ‘repeated’ contrasts, where applicable. In case of significant interaction effects involving ‘age’, patients with mild vs. moderate HL were subdivided in ‘older’ vs. ‘younger’ participants as defined by the respective HL-subgroup’s median age. We then repeated the respective rmANCOVAs (with ‘time since screening’ as covariate) as well as correlational analyses.

## 3. Results

We report (1) descriptive statistics for patients with mild vs. moderate HL, followed by (2) correlational analyses of (a) baseline values with SC and (b) baseline values with change in SC following the HA fitting, completion of the auditory training, and ending of the intervention. Next, we report (3) rmANCOVAs and post hoc analyses that examine change in SC across timepoints and noise interference levels, and explore the effects of age through second-line rmANCOVAs and correlational analyses for ‘young’ vs. ‘old’ participants, where applicable.

### 3.1. Descriptive Values for Patients with Mild or Moderate Hearing Loss

Table 2 provides an overview of descriptive values for participants with mild vs. moderate HL. Whilst participants’ levels of perceived stress (PSQ) and anxiety or depression (HADS) were low, general psychological- (ISR) and tinnitus-related distress (TQ, THI, and TFI) levels were mildly elevated. The descriptive values at screening are listed in Table 1 (see also [27]). Compared to participants with mild HL, participants with moderate HL had higher SC difficulties in silence at at 55 and 65 dB. For the psychological variables, no between-group differences emerged.

### 3.2. Correlational Analyses of Baseline Values with SC per Noise Interference Condition for Patients with Mild or Moderate Hearing Loss

Table 3 reports correlations between baseline values and patients’ SC for each noise interference condition. At 0 dB noise interference, higher SC difficulties were associated with older age, and higher TFI-measured TRD in patients with mild HL, but lower TQ-measured TRD in patients with moderate HL. At 55 dB noise interference, higher SC difficulties were associated with higher TRD in patients with mild, but not moderate HL. At 65 dB noise interference, higher SC difficulties were associated with higher TFI-measured TRD in patients with mild, but not moderate HL.

### 3.3. Correlational Analyses of Baseline Values with Change in SC per Noise Interference Condition for Patients with Mild or Moderate Hearing Loss

Table 4 reports associations between baseline values and change in SC for the (stability of) effects of the HAs and auditory training per noise interference condition.

At 0 dB noise interference, higher HA-related improvements were associated with (a) older age and lower perceived stress in patients with mild HL, and (b) older age and lower TQ-measured TRD in patients with moderate HL. Although the auditory training component did not significantly contribute to SC improvement over time (see Section 3.4), improvements were nonetheless with higher TRD and perceived stress in patients with mild but not moderate HL at baseline. Higher stability of the combined effect was associated with lower TRD, perceived stress, anxiety, depression, and psychological distress in patients with mild but not moderate HL.

At 55 dB noise interference, higher HA-related improvements were associated with higher TFI-measured TRD in patients with mild- and lower THI-measured TRD in patients with moderate HL.

At 65 dB noise interference, no significant associations emerged.

### 3.4. Terzo^©^ Hearing Therapy and Change in SC across Timepoints and Noise Interference Conditions

**Figure 2:**
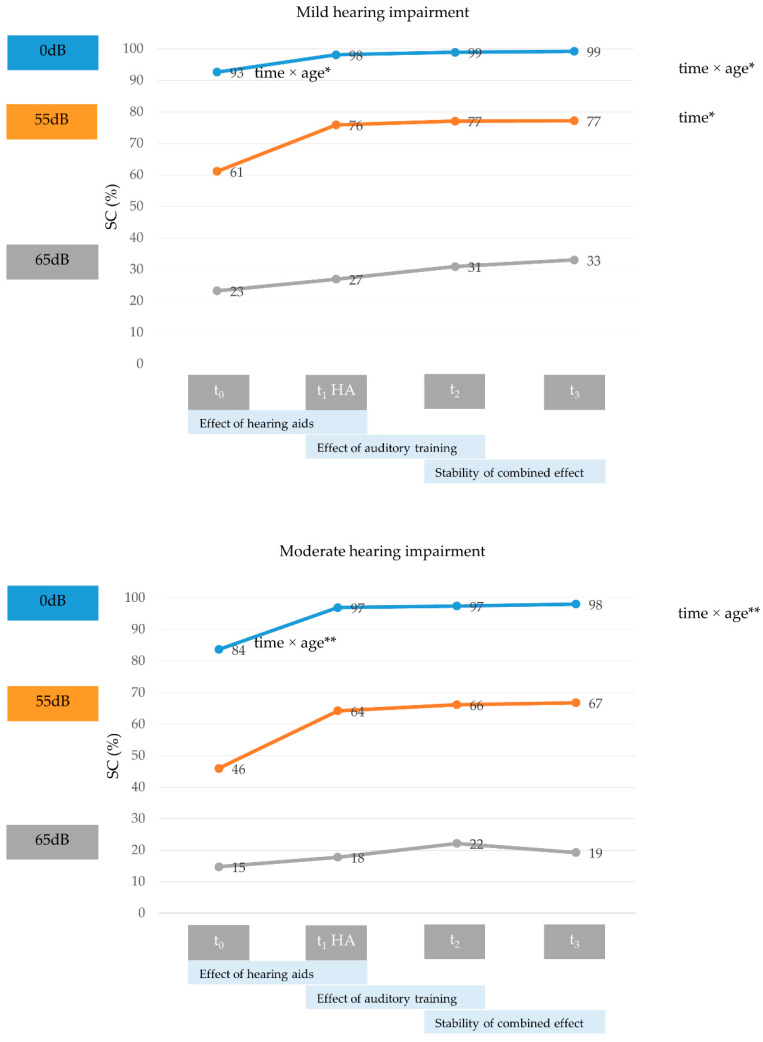
Effects of Terzo^©^ hearing therapy on speech comprehension. At 0 dB noise interference, the intervention yielded significant time × age interaction effects for patients with mild and moderate hearing loss. At 55 dB noise interference, the intervention yielded a significant main effect of time for patients with mild but not moderate hearing loss. HA = hearing aid fitting, SC = speech comprehension, * = *p* < 0.05; ** = *p* < 0.01.

Figure 2 illustrates the effects of Terzo^©^ hearing therapy on SC across all noise interference conditions for patients with mild or moderate HL.

At 0 dB noise interference, the intervention yielded significant time × age interaction effects for patients with mild (*F*_time × age_ [2.06,212.11] = 3.25, *p* < 0.05, *η_p_²* = 0.03, small effect) and moderate HL (*F*_time × age_ [1.29,49.02] = 6.99, *p* < 0.01, *η_p_²* = 0.16, large effect), both of which appeared to be driven by the time × age interaction effects of the HAs (post hoc analyses: patients with mild HL: t_0-_t_1_: *F*_time × age_ [1,103] = 4.42, *p* < 0.05, *η_p_²* = 0.04, small effect; patients with moderate HL: t_0_–t_1_: *F*_time × age_ [1,38] = 9.21, *p* < 0.01, *η_p_²* = 0.20, large effect).

At 55 dB noise interference, the intervention yielded a significant main effect of time for patients with mild (*F*_time_ [2.51,258.86] = 4.04, *p* < 0.05, *η_p_²* = 0.04, small effect) but not moderate HL. Post hoc analyses revealed no significant change but a weak trend (*p* = 0.06) for the t_0_–t_1_ effect of HAs.

#### Follow-Up Analyses Investigating the Influence of Age on the Effects of Terzo^©^ Hearing Therapy on SC at 0 dB Noise Interference 

To further investigate the time × age interaction effect on patients’ SC in silence, correlational analyses revealed an association between higher age and higher HA-related improvement in patients with mild (*r* = −0.21, *p* < 0.05) and moderate HL (*r* = −0.30, *p* < 0.05).

We further conducted two (patients with mild vs. moderate HL) × two (young (mild: <58.50; moderate: <63.50) vs. old) additional rmANCOVAs which found that, for patients with both mild and moderate HL, significant medium- and large-sized main effects of time emerged for older but not younger individuals. Post hoc analyses revealed significant improvements between t_0_ and t_1_ suggesting that, in silence, HAs are more beneficial for older patients than for younger ones (Patients with mild HL: total: *F*_time_ [1.15,59.64] = 7.50, *p* < 0.01, *η_p_²* = 0.13, medium effect; t_0_-t_1_: *F*_time_ [1,52] = 8.05, *p* < 0.01, *η_p_²* = 0.13, medium effect; Patients with moderate HL: Total: *F*_time_ [1.33,23.96] = 12.72, *p* < 0.01, *η_p_²* = 0.41, large effect; t_0_-t_1_: *F*_time_ [1,18] = 14.32, *p* < 0.01, *η_p_²* = 0.44, large effect).

Moreover, correlational analyses for younger vs. older patients with mild vs. moderate HL showed that in patients with mild, but not moderate HL, older (*r* = −0.29, *p* < 0.05) but not younger patients (*r* = 0.24, not significant; *z* = −2.95, *p* < 0.01) showed an association between higher HA-related improvement and higher TFI-measured TRD.

## 4. Discussion

The present study investigated the effects of Terzo^©^ hearing therapy for patients with mild (*n* = 124) and moderate (*n* = 53) HL on SC at four timepoints: after screening (t_0_; without HAs), after HA fitting (t_1_), after additional auditory training (t_2_), and at 70-day follow-up (t_3_) for three noise interference conditions (0 dB, 55 dB, and 65 dB). The intervention comprised binaural DSL_child_-algorithm-based HA fitting and 14 days of daily, CD-enhanced auditory training exercises.

Compared to patients with mild HL, patients with moderate HL were significantly older and had higher SC difficulties in silence and under noise interference—yet comparable, mildly elevated, levels of TRD. These findings are in agreement with previous studies that reported positive associations between age and HL [44] as well as SC difficulties in patients with chronic tinnitus [7]. Age-related psychophysiological changes that are associated with HL are common [45] and may extend to SC difficulties, although the underlying mechanisms are likely complex [12,46,47,48,49].

### 4.1. Patients with Mild Hearing Loss

#### 4.1.1. Speech Comprehension at Baseline

In patients with mild HL, lower SC was associated with (a) higher TFI-measured TRD at 0 dB noise interference; (b) higher anxiety and psychological distress, as well as higher TQ-THI- and TFI-measured TRD at 55 dB noise interference in this subgroup only; and (c) higher TFI-measured TRD at 65 dB noise interference in this subgroup only.

Against the backdrop of overall mildly elevated TRD levels [27], the here-observed associations emphasize the importance of psychological influences on SC beyond the impact of peripheral HL alone [7], possibly through interacting affective or cognitive routes [50,51,52,53]. For example, difficulties with peripherally mediated HL or, relatedly, SC, may exacerbate individuals’ anxiety [54,55], part of which may be attributed to the tinnitus symptomatology and experienced as TRD.

Interestingly, an association between TRD and SC emerged only in patients with mild but not moderate hearing loss. This finding elucidates the importance of conjointly considering the degree of HL and psychological distress when operationalising SC success or planning HA fittings [56]. Future studies ought to investigate data reflecting larger variance in HL, SC, and psychological distress in order to delineate respective contributions.

#### 4.1.2. Change in Speech Comprehension with Treatment

Higher HA-related improvements of SC were associated with (a) older age and lower perceived stress at 0 dB noise interference, and (b), in this subgroup only, higher TFI-measured TRD at 55 dB noise interference.

In line with existing recommendations [10], this finding adds to the recommendations for HAs as a frontline intervention for individuals with peripheral hearing loss and co-occurring SC difficulties. It has been suggested that the TFI might reflect more audiological facets of TRD whilst other common self-report measures, such as the THI or TQ, may reflect broader psychological distress that may then be attributed to the tinnitus symptom [57]. Thus, multimodal conceptualisations of hearing loss, fluctuations in SC, and psychological influences in the context of chronic tinnitus symptomatology are warranted and need joint interpretation.

Despite the overall low levels of psychological distress, the association between the beneficial effects of HAs on SC and lower perceived stress suggests the influence of psychological factors on the effectiveness of audiological interventions [58,59] and vice versa [60,61]. Whilst larger studies ought to investigate this hypothesis in a sample with higher distress variation, individual, psycho-audiological problem conceptualisations and tailored, multimodal intervention strategies appear warranted to maximise intervention benefits—even within ‘classical’ audiological domains.

In patients with mild HL, higher auditory training-related improvements were associated with higher TRD and perceived stress.

Although the auditory training did not significantly improve patients’ SC beyond the effects of the Terzo^©^ HA fitting (cf. below), its effect was associated with higher levels of psychological distress at baseline. Against the backdrop of our predecessor paper, which reported overall psychological benefits following hearing therapy [27], the present finding may suggest a closer link between the auditory training component and psychological, rather than audiological, outcomes (SC). Given the strong association between HL and psychological distress [62,63], the auditory training component may benefit individuals with more centrally mediated, distress-related difficulties with hearing or SC in the context of mild HL. Future studies ought to stratify patients by degrees of HL and psychological distress to further investigate this hypothesis.

Moreover, higher stability of the combined effect was associated with lower TRD, perceived stress, anxiety, depressivity, and psychological distress at the baseline, again highlighting the potential importance of psychological contributors to the effects of the here-investigated hearing therapy on SC [7].

#### 4.1.3. Effects of Terzo^©^ Hearing Therapy

Terzo^©^ hearing therapy yielded a medium-sized, significant *time × age* interaction effect at 0 dB noise interference. This effect was driven by an age-dependent effect of HAs.

Exploratory additional analyses suggested a main effect of HAs for older but not younger patients with mild HL. Moreover, Terzo^©^ hearing therapy resulted in a small-sized, significant main effect of time at 55 dB noise interference that was driven by a trend effect of HAs.

This finding is in agreement with the literature emphasizing age in the process of HA fitting [64] and suggests that the here-investigated HA fitting significantly improved SC for chronic tinnitus patients with mild HL under conditions of little or no noise interference. The auditory training did not exert an additional effect on SC. In context of overall low psychological distress but significant associations between auditory training effects and (younger) age, possible effects of the auditory training for individuals with higher levels of psychological distress but comparable levels of HL must remain speculative at this point [27].

### 4.2. Patients with Moderate Hearing Loss

#### 4.2.1. Speech Comprehension at Baseline

In patients with moderate HL, higher SC was associated with higher TQ-measured TRD at 0 dB noise interference.

Although preliminary, the tinnitus-related or broader emotional distress might modulate executive control towards heightened processing of external stimuli in the context of moderate hearing loss, for example by means of altered executive control or allocation of attentional resources [65,66,67]. If replicated in future studies, audiological, cognitive, and emotional factors ought to be jointly assessed and individually interpreted in light of bidirectional effects that may be moderated by patients’ degree of hearing loss.

#### 4.2.2. Change in Speech Comprehension with Treatment

Higher HA-related improvements were associated with (a) lower TQ-measured TRD at 0 dB noise interference, and (b) lower THI-measured TRD at 55 dB noise interference.

In line with previous studies investigating psychological influences on successful HA use [68,69], psychological distress appears to potentially moderate HAs’ success in improving SC in individuals with moderate HL. The observed correlations may reflect the shared central processes between psychological distress and audiological performance [70]; however, research to this regard remains in its infancy.

#### 4.2.3. Effects of Terzo^©^ Hearing Therapy

Terzo^©^ hearing therapy yielded a large-sized, significant *time × age* interaction effect at 0 dB noise interference, which was driven by an age-dependent effect of HAs. Additional exploratory analyses again suggested the main effect of HAs for older but not younger patients at 0 dB noise interference.

## 5. Limitations

The current study has important limitations, most notably the absence of control group analyses and the non-stratification of included patients by psychological distress. Importantly, owing to the study’s inclusion and exclusion criteria, the current results cannot be generalised to individuals with severe hearing loss.

## 6. Conclusions

In summary, the results of the present study revealed benefits of Terzo^©^ HA fitting on SC for patients with chronic tinnitus and mild or moderate HL under conditions of little or no noise interference. At medium-level noise interference, HAs benefit patients’ SC in context of mild but not moderate HL. Psychological variables appear to interact with the degree of peripherally mediated HL in influencing both patients’ SC and HA-related benefits, however inconclusively so. Overall, low psychological distress rates prohibited further examination of affective–cognitive influences; thus, interactions of emotional distress and intervention benefits (including the duration of HA-use) remain to be investigated in future studies.

## Figures and Tables

**Table 1:** Sociodemographic data and patient characteristics (*N* = 177).

		n	%
Education			
	Completed junior apprenticeship	72	40.7
	Completed senior apprenticeship	40	22.6
	University degree	60	33.9
	Other	4	2.3
Employment ‘yes’		105	59.3
Relationship status			
	Single	25	14.1
	Married	114	64.4
	Divorced	27	15.3
	Widowed	10	5.6
Duration of tinnitus			
	<0.5 year	5	2.8
	0.5–1 year	9	5.1
	1–2 years	23	13.0
	2–5 years	24	13.6
	>5 years	107	60.5
Tinnitus onset			
	Gradual	92	52.0
	Sudden	73	41.2
Frequency			
	Very High	37	20.9
	High	104	58.8
	Middle	32	18.1
	Low	3	1.7
Past psychotherapy ‘yes’		53	29.9
Use of hearing aid ‘yes’		53	31.5

**Table 2:** Descriptive indices for patients with mild (*n* = 124) vs. moderate hearing loss (*n* = 53). SC = speech comprehension; TQ = Tinnitus Questionnaire; THI = Tinnitus Handicap Inventory; TFI = Tinnitus Functional Index; PSQ = Perceived Stress Questionnaire: HADS_a = Hospital Anxiety and Depression Scale; anxiety; HADS_d = depression; ISR = ICD-10 Symptom Rating; *d* = effect size; ** *p* < 0.01; *** *p* < 0.001.

	Mild (*n* = 124)		Moderate (*n* = 53)				
Baseline	*M*	*SD*	*M*	*SD*	Difference	*t*(df)	*d*
Age	58.46	7.17	62.35	7.51	−3.89	*t*(174) = −3.24 **	−0.54
Hearing ability	33.47	5.08	45.62	3.88	−12.15	*t*(126.99) = −17.31 ***	−2.56
SC 0 dB	92.89	9.55	83.73	13.78	9.17	*t*(71.071) = 4.33 ***	0.84
SC 55 dB	61.41	15.06	47.25	13.83	14.15	*t*(170) = 5.76 ***	0.96
SC 65 dB	24.30	16.81	15.39	14.03	8.91	*t*(170) = 3.33 **	0.56
TQ	33.32	15.76	33.23	17.03	0.10		
THI	33.39	21.96	32.04	22.49	1.35		
TFI	39.58	20.35	40.48	20.77	−0.90		
PSQ	30.05	16.65	32.86	23.89	−2.82		
HADS_a	6.35	4.07	7.43	4.75	−1.09		
HADS_d	4.98	4.38	6.15	5.25	−1.17		
ISR	0.61	0.50	0.74	0.59	−0.13		

**Table 3:** Pearson’s *r* correlation coefficients for baseline values and SC across three noise interference conditions for patients with mild vs. moderate hearing loss. SC = speech comprehension; TQ = Tinnitus Questionnaire; THI = Tinnitus Handicap Inventory; TFI = Tinnitus Functional Index; PSQ = Perceived Stress Questionnaire: HADS_a = Hospital Anxiety and Depression Scale; anxiety; HADS_d = depression; ISR = ICD-10 Symptom Rating; * *p* < 0.05; ** *p* < 0.01.

	Mild (*n* = 124)	Moderate (*n* = 53)		Mild (*n* = 124)	Moderate (*n* = 53)		Mild (*n* = 124)	Moderate (*n* = 53)	
Noise Interference	SC 0 dB	SC 0 dB	*z*	SC 55 dB	SC 55 dB	*z*	SC 65 dB	SC 65 dB	*z*
Age	−0.25 **								
Hearing ability	−0.37 **	−0.35 *		−0.29 **	−0.42 **		−0.25 **		
TQ	−0.04	0.37 **	2.45 *	−0.20 *	0.21	−2.39 *			
THI				−0.21 *	0.23	−2.62 **			
TFI	−0.20 **	0.23	−2.57 *	−0.31 **	0.19	−2.99 **	−0.20 *	0.23	−2.56 *
PSQ									
HADS_a				−0.22 *					
HADS_d									
ISR				−0.28 **					

**Table 4:** Pearson’s *r* correlation coefficients for baseline values and change in SC across 0 dB and 55 dB noise interference conditions for patients with mild vs. moderate hearing loss. In the 65 dB noise interference condition, no effects emerged. SC = speech comprehension; TQ = Tinnitus Questionnaire; THI = Tinnitus Handicap Inventory; TFI = Tinnitus Functional Index; PSQ = Perceived Stress Questionnaire: HADS_a = Hospital Anxiety and Depression Scale; anxiety; HADS_d = depression; ISR = ICD-10 Symptom Rating; mod = moderate; * *p* < 0.05; ** *p* < 0.01.

Change in SC	t_0_–t_1_			t_1_–t_2_			t_2_–t_3_			t_0_–t_1_			t_1_–t_2_			t_2_–t_3_		
Effects	Hearing Aids			Auditory Training			Stability			Hearing Aids			Auditory Training			Stability		
Noise Interference	0 dB			0 dB			0 dB			55 dB			55 dB			55 dB		
Hearing loss	Mild *n* = 124	Mod *n* = 53	*z*	Mild *n* = 124	Mod *n* = 53	*z*	Mild *n* = 124	Mod *n* = 53	*z*	Mild *n* = 124	Mod *n* = 53	*z*	Mild *n* = 124	Mod *n* = 53	*z*	Mild *n* = 124	Mod *n* = 53	*z*
Age	−0.21 *	−0.30 *																
Hearing ability		−0.37 **																
TQ		0.36 *		−0.26 **	0.22	−2.71 **	0.34 **	0.05										
THI				−0.27 **	0.10	−2.08 *	0.31 **	0.02		−0.09	0.29 *	−2.23 *						
TFI				−0.30 **	0.16	−2.52 *	0.31 **	0.08		−0.19 *	0.27	−2.68 **						
PSQ	0.23 *			−0.28 **	0.06		0.20 *	0.18										
HADS_a							0.21 *	0.20										
HADS_d							0.19 *	0.21										
ISR							0.25 *	0.16										

## Data Availability

As per Charité Universitaetsmedizin Berlin’s ethics committee, unfortunately, we cannot make the data public without restrictions because we did not obtain patients’ consent to do so at the time. Nevertheless, interested researchers can contact the directorate of the Tinnitus Centre Charité Universitaetsmedizin Berlin with data access requests (birgit.mazurek@charite.de).
